# GIFtS: annotation landscape analysis with GeneCards

**DOI:** 10.1186/1471-2105-10-348

**Published:** 2009-10-23

**Authors:** Arye Harel, Aron Inger, Gil Stelzer, Liora Strichman-Almashanu, Irina Dalah, Marilyn Safran, Doron Lancet

**Affiliations:** 1 Department of Molecular Genetics, Weizmann Institute of Science Rehovot 76100, Israel; 2 Department of Biological Services (Bioinformatics Unit), Weizmann Institute of Science, Rehovot 76100, Israel

## Abstract

**Background:**

Gene annotation is a pivotal component in computational genomics, encompassing prediction of gene function, expression analysis, and sequence scrutiny. Hence, quantitative measures of the annotation landscape constitute a pertinent bioinformatics tool. GeneCards^® ^is a gene-centric compendium of rich annotative information for over 50,000 human gene entries, building upon 68 data sources, including Gene Ontology (GO), pathways, interactions, phenotypes, publications and many more.

**Results:**

We present the GeneCards Inferred Functionality Score (GIFtS) which allows a quantitative assessment of a gene's annotation status, by exploiting the unique wealth and diversity of GeneCards information. The GIFtS tool, linked from the GeneCards home page, facilitates browsing the human genome by searching for the annotation level of a specified gene, retrieving a list of genes within a specified range of GIFtS value, obtaining random genes with a specific GIFtS value, and experimenting with the GIFtS weighting algorithm for a variety of annotation categories. The bimodal shape of the GIFtS distribution suggests a division of the human gene repertoire into two main groups: the high-GIFtS peak consists almost entirely of protein-coding genes; the low-GIFtS peak consists of genes from all of the categories. Cluster analysis of GIFtS annotation vectors provides the classification of gene groups by detailed positioning in the annotation arena. GIFtS also provide measures which enable the evaluation of the databases that serve as GeneCards sources. An inverse correlation is found (for GIFtS>25) between the number of genes annotated by each source, and the average GIFtS value of genes associated with that source. Three typical source prototypes are revealed by their GIFtS distribution: genome-wide sources, sources comprising mainly highly annotated genes, and sources comprising mainly poorly annotated genes. The degree of accumulated knowledge for a given gene measured by GIFtS was correlated (for GIFtS>30) with the number of publications for a gene, and with the seniority of this entry in the HGNC database.

**Conclusion:**

GIFtS can be a valuable tool for computational procedures which analyze lists of large set of genes resulting from wet-lab or computational research. GIFtS may also assist the scientific community with identification of groups of uncharacterized genes for diverse applications, such as delineation of novel functions and charting unexplored areas of the human genome.

## Background

In the quest for revealing the function of DNA sequences, scientists have used a variety of approaches, from molecular techniques targeting specific genes, to systematic analyses of thousands of functional units encompassed by the transcriptome, proteome, and metabolome. This heterogeneous mass of knowledge is time-dependent, with new information constantly arising from a variety of sources. Thus, a quantitative tool for assessing annotation depth is important for directing ongoing research and for analyzing the emerging results. Efforts in this field have included the Genome Annotation Scores (GAS) algorithm [1], which demonstrates a quantitative methodology of assigning annotations scores at the whole genome level, the GO Annotation Quality (GAQ) score, which gives a quantitative measure of GO annotations [2], and the Gene Characterization Index (GCI), which scores the extent to which a gene's functionality is described, based largely on the quantification of human perception, and applied only to protein-encoding genes [3]. We now introduce the GeneCards Inferred Functionality Scores (GIFtS) tool [4], which utilizes the wealth of gene annotation within GeneCards [5] to quantify the degree of functional knowledge about >50,000 GeneCards entries. GeneCards is a comprehensive gene-centric compendium of annotative information about human genes, automatically mined from nearly 70 data sources [6-12]. Thus, GIFtS can provide quantitative annotation estimates for a very large number of genes, and at a significant depth, made possible by the exploitation of dozens of annotation resources.

## Results

### GIFtS definition and applications

**Figure 1:**
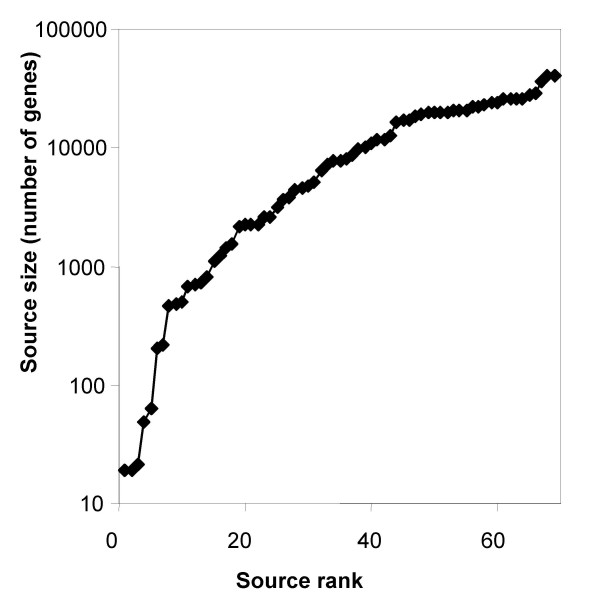
**Source size**. The number of human gene entries in each one of the sources contributing to the GIFtS score. Sources are shown by their rank according to their size (see additional file 1: Table S1). A source's size is defined to be the number of GeneCards entries containing annotations from this source.

We devised the GeneCards Inferred Functionality Score (GIFtS) which allows a quantitative assessment of a gene's annotation status, with potential relevance to the degree of relevant functional knowledge. A GIFtS value for a gene is defined as the number of GeneCards sources, out of a total of 68 (see additional file 1: Table S1), that include information about this gene (see Methods). Data sources have heterogeneous sizes, as estimated by the number of human gene entries for which the source contains information (Fig. 1), having an average of 11,404 ± 10,970 entries per source. One of GeneCards' main aims is to incorporate overlapping sources, and perform integration of data for different annotation fields. Considerable attention is also directed to conflicts among sources, one clear example being the GeneLoc [8] member of the GeneCards suite, which handles conflicts in genomic coordinates from Ensembl [13] and NCBI [14]. The overlap problem is particularly applicable for data extracted from genome-wide sources such as Entrez Gene, Ensembl, GO [15], UniProt [16] and InterPro [17] which are all closely linked and share some of the information presented, which may introduce a degree of redundancy.

To study the effect of overlap between sources, we generated a pairwise overlap matrix for all 68 data sources (see additional file 2, 3, 4: Tables S2, S3 and Fig. S1-A). For this we utilized a parameter (see Methods) which assesses the overlap by gene set sharing. The rational is that if, for example, all information about a set of genes, such as microRNAs, is imported from one source to another, it will result in an overlap in gene sets. Based on the overlap matrix, we proceeded to select the 20 source pairs with the highest overlap (≥0.8), and eliminated (typically) one source of each pair. Fig. 1S-B (see additional file 3: Fig.S1) shows that the exclusion of overlapping sources has only a minor effect on the overall shape of the distribution, hence, by inference, on the relative positions of the majority of genes on the GIFtS scale. This may suggest that having an overlap among sources does not impose an adverse effect on proposed functionality scale. While such a finding could be interpreted as suggesting over-representation of information items, we believe that source redundancy also has its advantages, as pointed out below.

We also asked whether overlap might exert an advantageous effect. For this, we comparatively analysed the GCI scale with 6 data sources, i.e. having a lower (but not negligible) degree of source overlap, and GIFtS, which has 68 sources and a considerably higher extent of source overlap. Focusing on the 489 genes with highest functionality scores in GCI, all having the same score of 10.0, we note that the same genes span a rather broad range of GIFtS values, from 51 to 84 (see additional file 5: Fig. S2-A). Thus, for the most well-annotated genes, the GIFtS scale displays a fine resolution not seen in GCI. We suspect that this may actually arise from the diversity of overlapping sources in GIFtS. That this is the case is tentatively supported by the fact that when eliminating the 21 smallest-size sources (below source size of 2200, see characterization of data sources by GIFtS, and additional file 1: Table S1) the spanned score range for the top 500 GeneCards genes diminishes appreciably, from 68-84 (span of 16 units) to 92-100 (span of 8 units) (see Methods for scale shift details). When performing a similar analysis for 1961 low-annotation genes, all having a GCI score of exactly 2.2, a wide range of GIFtS scores was seen again, between 2 and 34 (see additional file 5: Fig. S2-B), suggesting that the enhanced resolution afforded by the larger number of sources is not unique to top-scoring genes.

GIFtS is fully implemented in GeneCards. Every GeneCards gene entry is marked with its GIFtS value. Also provided on the GeneCards homepage is a capacity for obtaining a random gene with a specified GIFtS score within a given range, constituting a useful tool for browsing the human gene annotation landscape. Linked from the GeneCards homepage is the expanded GIFtS tool, which affords additional functionalities such as: a) retrieving a list of genes within a specified range of GIFtS value, thereby obtaining random genes with more detailed GIFtS specifications; b) obtaining a list of sources that did not comprise data for a selected gene, thereby facilitating further characterization for genes of interest, and potentially useful for understanding poorly researched genes; c) obtaining the GIFtS value of a selected gene, which results in an annotation indicator graphically superimposed on the overall distribution graph; and d) displaying GIFtS distributions and statistics.

### GIFtS distributions

**Figure 2:**
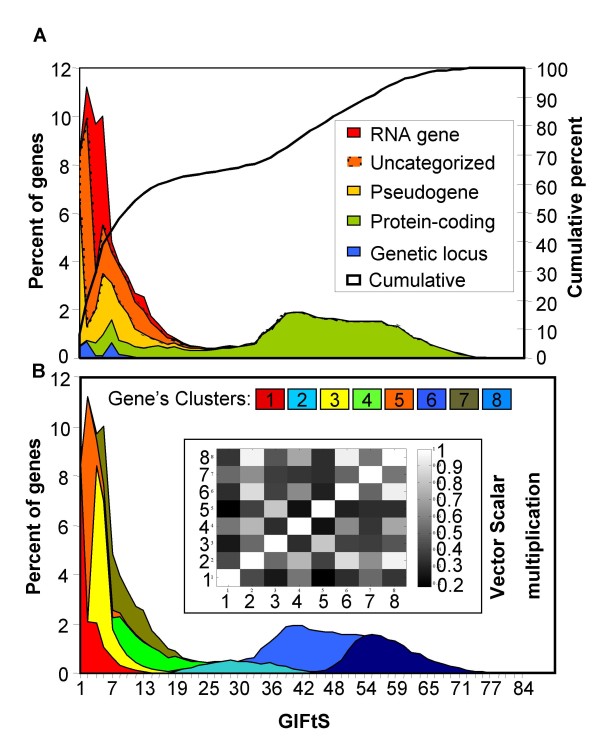
**Distribution of GeneCards Inferred Functionality Scores (GIFtS)**. (A) Dissection of GIFtS by gene categories. (B) Clustering analysis of sources of the GIFtS tool (with highest significance for cluster 6, T-test for difference of means, p < 0.05). Inner frame presents correlation between clusters (highest correlation -1) calculated by scalar-multiplication of unit vectors of cluster centers.

To explore the annotation landscape of the human genome, we analyzed the distribution of GIFtS for all GeneCards entries (Fig. 2). The bimodal shape of the GIFtS distribution suggests a division of the human gene repertoire into two main groups, based on the degree of annotation. Dissecting the curve according to GeneCards gene categories (Fig. 2A), we find that the high-GIFtS peak consists almost entirely of protein-coding genes, while the low-GIFtS peak consists of genes from all of the categories (see examples in Table 1). It should be pointed out that low GIFtS genes include not only those originally identified as predicted genes, e.g. CiiORFjjj (where ii is a chromosome number) and FAMxxx genes (family with sequence similarity), but also many genes that are not easily defined by their symbols, such as non protein-coding genes.

**Table 1:** GIFtS values for selected genes.

	**Gene_Symbol**	**Category**	**Gene_Description**	**HGNC Approval**	**GIFtS**
1	TP53	Protein-coding	Tumor protein p53	Yes	84

2	SOD1	Protein-coding	Superoxide dismutase 1, soluble	Yes	82

3	NFKB1	Protein-coding	Nuclear factor of kappa light polypeptide gene enhancer in B-cells 1	Yes	78

4	RAC2	Protein-coding	Ras-related C3 botulinum toxin substrate 2 (rho family, small GTP binding protein Rac2)	Yes	76

5	IL2	Protein-coding	Interleukin 2	Yes	73

6	BRCA1	Protein-coding	Breast cancer 1, early onset	Yes	72

7	CD4	Protein-coding	CD4 molecule	Yes	72

8	DLEU1	RNA-gene	Deleted in lymphocytic leukemia 1 (non-protein coding)	Yes	48

9	HMGB1L10	Pseudogene	high-mobility group box 1-like 10	Yes	31

10	GBAP	Pseudogene	Glucosidase, beta; acid, pseudogene	Yes	28

11	ABCC6P1	Pseudogene	ATP-binding cassette, sub-family C, member 6 pseudogene 1	Yes	9

12	MIRN1224	Uncategorized	MicroRNA 1224	Yes	7

13	SNORA11D*	RNA gene	Small nucleolar RNA, H/ACA box 11D	Yes	6

14	C14orf7*	Protein-coding	Chromosome 14 open reading frame 7	Yes	4

15	AASTH1*	Genetic locus	Allergic/atopic asthma related QTL 1	No	1

16	C6orf179*	Uncategorized	Chromosome 6 open reading frame 179	Yes	1

* Unknown genomic location.

Well studied genes are prominent at the top of the table. Lines 8-10 depict genes with unexpectedly high GIFtS values for their category.

We asked what might distinguish genes with extremely high GIFtS score (>75, right tail of the high-GIFtS peak) as compared to those at the top of the same peak (genes with GIFtS value of 51); we note that some sources are particularly enriched (>X8) in the former. Such sources include, for example, databases on Genetic Association (GAD), Cytogenetics in Oncology and Haematology (ATLAS), Human Gene Mutation (HGMD), drug response variations (PHARMGKB), news about biomedical research (DOCTOR'S GUIDE) genetic testing of inherited disorders (GENETESTS) and Human Genome Epidemiology (HUGE NAVIGATOR), all indicative of advanced stages in functional gene annotation.

We subsequently clustered the GeneCards entries according to the source combinations that provide their annotation (Fig. 2B). As an example, under the assumption of 8 clusters, the most distinct was cluster 6 (T-test for difference of means, p < 0.001), with contribution from the sources AlmaKnowledge Server (AKS, [18]), Expoldb [19] and GeneWiki [20]. Since these sources integrate data which is usually available for highly annotated genes (e.g. AKS uses text mining to find genes' interactions with chemical compounds and their relationships to diseases), it is conceivable that they appear in this unique cluster of high GIFtS. We see that different parts of the GIFtS distribution are dominated by different clustered patterns of annotation sources. Genes with high GIFtS tend to consist of data from the same source combination, as shown by the high degree of similarity among clusters of the high GIFtS peak (Fig. 2B, inset).

### Characterization of data sources by GIFtS

**Figure 3:**
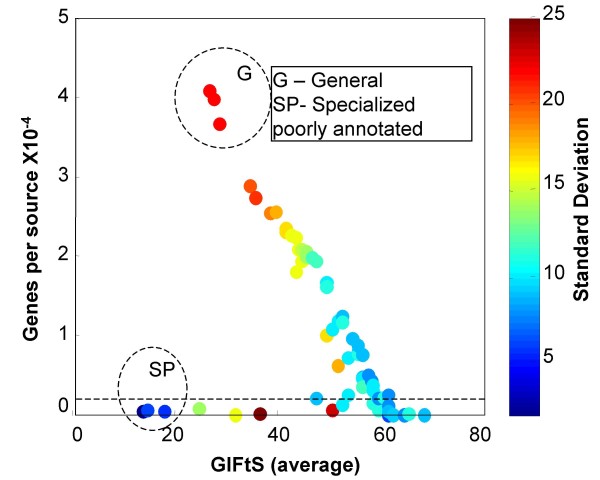
**Characterization of GeneCards sources by GIFtS statistics**. Characterization of GeneCards sources by GIFtS statistics. Each point represents all genes within a given annotation source (circles below dashed line were excluded for the production of GIFtS reduced by 21 lowest ranked sources, see additional file 1: Table S1). Generating the GIFtS distribution for each source achieved higher resolution (e.g. Fig. 4).

GIFtS is also useful for characterizing and comparing different database sources. Plotting source size against GIFtS values, an inverse correlation was found for GIFtS>25 (Fig. 3). While the top of the graph (labeled G) comprises general sources pertinent to a large number of different genes (e.g. NCBI Entrez Gene [14], Ensembl [13]) the lower end of this curve includes sources that specialize in a small number of highly annotated genes (euGenes [21], HGMD [22]). A separate cluster (SP) arises from another class of specialized sources (e.g. miRBase [23], IMGT [24]) whose genes tend to be poorly studied. The standard deviation of GIFtS values tends to be higher for the larger, high-GIFtS sources, reflecting annotation heterogeneity (represented by colors in Fig. 3).

**Figure 4:**
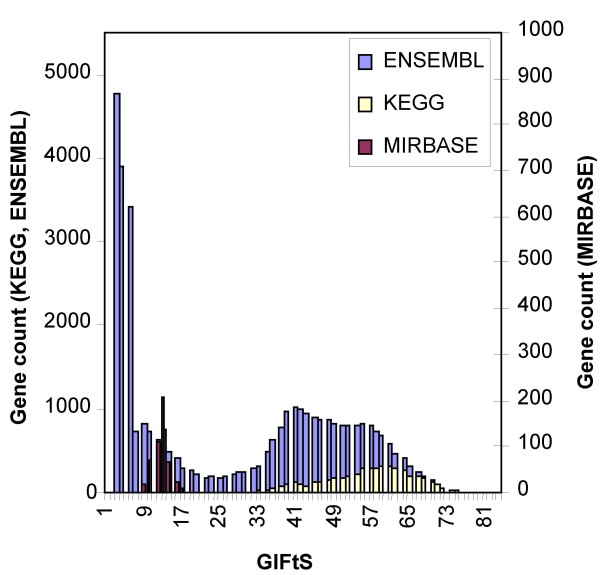
**GIFtS fingerprint**. Distribution of GIFtS values for 3 selected sources produced three different archetypes representing sources encompassing: well studied genes (KEGG), all genes (Ensembl), genes which are poorly studied (miRBase).

To achieve a higher resolution of sources analysis, we calculated GIFtS distributions for all sources. Three typical prototypical distributions are shown in Fig. 4. The first (illustrated by Ensembl) is genome-wide sources whose GIFtS distribution conforms with the one seen when all sources are combined together for all GeneCards entries. The second relates to sources, characterizing already known genes, whose genes are mainly found in the high GIFtS peak, illustrated by KEGG [25]. The third includes sources, characterizing new and unusual genes, whose genes are mainly found in the low GIFtS peak, as exemplified by miRBase.

### GIFtS facilitates the analysis of experimental genes sets

Inspecting the level of annotation for a list of genes generated by experiments can supply additional insight about the data, and may facilitate research by suggesting targets for further study. As an example, we have generated the GIFtS distribution for genes whose expression was significantly altered *vs *controls in 3 different published experiments (see additional files 6, 7: Fig. S3-A and Table S4). As seen, different datasets show somewhat different distributions, suggesting better or worse typical prior knowledge about the constituent genes. In each of the sets, well studied genes in the high GIFtS region may suggest a higher probability for being associated with the relevant biological change, while genes in the low GIFtS tail may necessitate further earmarked functional studies. In parallel, we have performed a similar analysis for three sets of genes derived from GeneCards searches with keywords with different expected level of annotation, and showed that indeed the GIFtS distribution can capture such annotation differences that result from different depths of experimental evidence (see additional files 6, 8: Fig. S3-B, Table S5).

**Figure 5:**
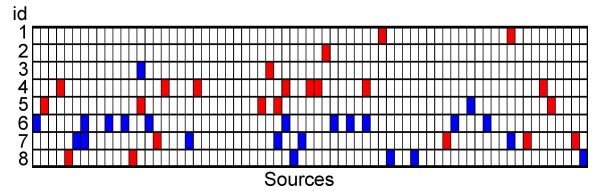
**Sources enrichment analysis**. The pattern of each of the sources enrichment for each of eight gene sets expressed exactly in two tissues (see additional file 10: Table S7) was calculated based on the significance (p < 0.07) of each of the source enrichments in each set. Blue and red squares indicate over or under representation of source in set, respectively (see additional file 9: Table S6). Ids indicate co-expression in: 1. Brain and Muscle; 2. Brain and Pancreases; 3. Brain and Prostate gland; 4. Brain and Thymus; 5. Lung and Kidney; 6. Lung and Spleen; 7. Muscle and Bone-Marrow; 8. Pancreases and Muscle.

The power of GIFtS in aiding experimental research so as to achieve deeper understanding of a gene sets is further highlighted by a source-depletion analysis. For this we utilized, as an example, 8 sets of genes expressed in exactly two normal human tissues (see Methods) derived from microarray experiments previously performed in our laboratory [12]. We asked what might be recommended for further scrutiny based on GIFtS-related evaluation of the information available about each of these gene sets. Within the GIFtS database, each gene in every one of the eight sets is characterized by a different pattern of sources from which information is available. We compute a statistic stemming from the set-average of such annotation values, and identify the sources with the least information for the given gene set (Fig. 5). Such paucity of representation of a given source in a gene set (see additional file 9: Table S6) can point to new directions within the researcher's set-specific studies. For example, under-representation of the source ASD [26] in the genes expressed in pancreas and muscle indicates lack of sufficient information about alternative splicing; under-representation of KEGG [25] in the genes expressed in the brain and muscle, similarly indicates a need to seek more information on pathways related data; under-representation of SwissProt [27] and InterPro [17] in the genes expressed in the brain and thymus, indicates lack of sufficient information in the protein level, including protein domains.

Another feature that assists in gauging biological results as contained within GeneCards has been introduced in the new GeneCards version 3 beta [28]. The user can select gene category or type (such as protein-coding genes or RNA genes) as well as GIFtS when browsing the genome by looking for a random gene or performing an advanced search.

### Time-Evolution of human genomic knowledge

**Figure 6:**
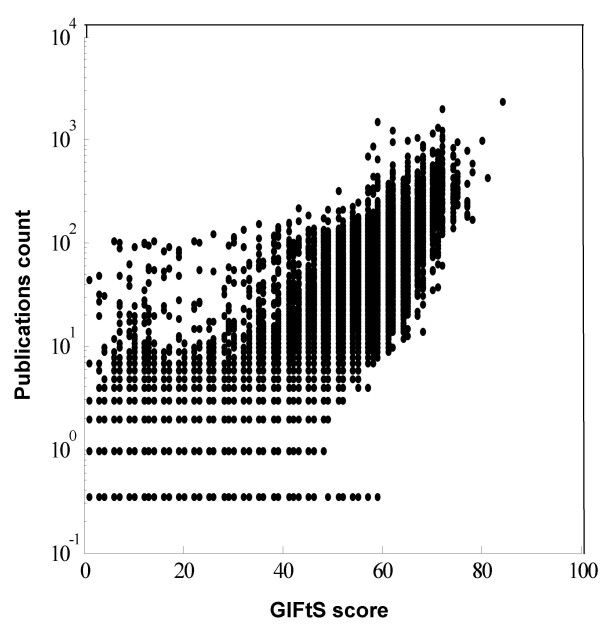
**Correlation of publication count and GIFtS**. Correlation between GIFtS scores and the count of publications to each GeneCards gene.

**Figure 7:**
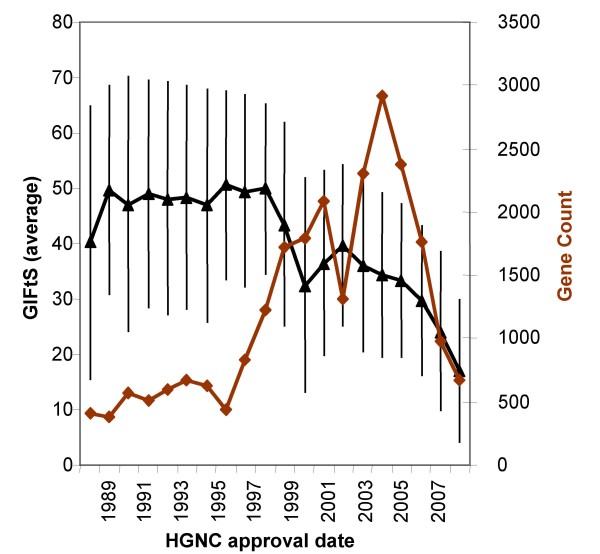
**Time-dependence of annotation levels**. Correlation between Human Gene Nomenclature Committee (HGNC) approval dates [39] and GIFtS average (bars represent standard deviation).

We suggest that GIFtS scores reflect the degree of known functionality for a gene, i.e. its annotation depth. One plausible parallel measure is the number of publications for a given gene, as found in its GeneCards entry with integration of gene-publication links from seven different sources [14,16,18,29-31]. Indeed, there is ample correlation (for GIFtS>30) between GIFtS and publications count, which are not part of the standard GIFtS score (Fig. 6), but the lack of perfect correlation, comprising a small group of 60 genes (of which only one is protein coding) with GIFtS<13 and publications count>= 10, suggests that GIFtS provides additional information. Another GIFtS correlation is with HGNC gene symbol approval date [30], with higher GIFtS genes tending to be older (Fig. 7). Genes with symbols approved before 1999 show a plateau GIFtS value of nearly 50, conforming to the approximate median of genes in the higher peak of the distribution of GIFtS for all GeneCards entries. This is statistically significantly different relative to genes approved after 1999, which show a declining trend to a low of GIFtS of ~15 by 2007 (T-test for difference of means, p < 0.001).

### GIFtS subsectioning and weighting

In its simplest embodiment, used throughout this paper, the GIFtS tool regards each source as a unitary entity, irrespective of content. However, we have also installed improvements within the GIFtS web tool [4], that include experimenting with increased resolution by using sub-sectioning of data sources and adjusting scores based on the presence or absence of detailed annotations within a source. In addition we have introduced weights related to the quantitative aspects of annotations items, enabling better evaluation of the data relevant to annotation levels. Sub-sectioning was performed on data from a pivotal bioinformatics source for proteins data, SwissProt [27], whereby a user may request scores based on the presence or absence of detailed annotations within this source (see Methods). Weights were introduced for publications (based on article counts per gene) and for orthologs (based on the number of species for which orthologs are shown).

With the latter modification, it is possible to perform explorations in the domain of low-GIFtS genes as follows: the user can request a comprehensive list of genes with GIFtS scores in a pre-selected range, activating the orthologs and publications options. The output then indicates genes for which there are known orthologs and published articles, so as to facilitate the initiation of study of a subset of the low GIFtS gene list.

We note that annotation about low-GIFtS genes is often limited to high-throughput generic genome publications, a situation which may reduce the usefulness of the publication-adjusted scores. We have therefore screened our database for such generic publications by looking for articles correlated with many genes. We found that only 25 publications are linked to >1000 genes and that 51 publications are linked to >500 genes. Genes whose only publications belong to such a category, and that are also represented in a large fraction (say 50% or higher) of all such publications, contain GIFtS scores of ~1-2, highlighting them as extremely low GIFtS genes. For genes in the higher GIFtS realm, the publication adjustment is almost negligible.

## Discussion

GIFtS provides a quantitative tool for assessing annotation depth of every gene in the human genome based on GeneCards' rich compendium of information sources. Three previously published relevant efforts focus only on protein coding genes; two of them are mainly aimed at gene sets and utilize more limited information sources. The first, the Genome Annotation Scores (GAS) algorithm [1], is broader in scope, since it addresses all species present in SwissProt [27]. Using 5 major information areas, literature, curation, sequence, structure and experiment, it provides only an average annotation score for all protein-coding genes in a given species. The second tool, the GO Annotation Quality (GAQ) score [2] is a quantitative annotation measure based on the number, detail and evidence type of GO annotations available for a gene. GAQ uses only one information source, and in contrast to GIFtS, focuses on assessing the annotation for an entire set of gene entries. The third tool, the Gene Characterization Index (GCI) [3] assesses the characterization status of individual human genes using annotations from six information sources, combined with the count of articles per each gene based on data from SwissProt [27] and NCBI Entrez Gene [14]. It is based on an original concept, whereby the perception of human researchers about the annotation status of a subset of genes is used as training data for developing a procedure to assign scores to all human genes. GCI allows one to identify sets of low-scoring genes suited for experimental investigation as drug targets. GIFtS, on the other hand, offers a non-biased approach which does not involve human perception, scores all human genes (whether protein coding or not), and is based on a much larger set of data sources.

One of the obvious advantages of the GIFtS scoring method is its applicability to all genes, irrespective to type and annotation depth. In recent years, several studies have highlighted the important role of non protein-coding genes (such as RNA genes) in cellular function [32-34]. Therefore, it is important to develop bioinformatics tools such as GIFtS which are applicable to all types of genes. However, in a world of fleeting gene annotations, it appears that GCI and GIFtS are complementary to each other: while GCI contains about 33,000 entries, all defined as protein coding [3], GIFtS (via GeneCards) addresses only ~22,000 genes defined to be protein coding (based on uniprot, refseq, and/or ensembl evidence), along with another ~10,000 non-protein-coding genes (categorized as pseudogenes, RNA genes, or disorder loci), and another ~10,000 which are still uncategorized). Future scrutiny could help resolve this dichotomy.

The usability of GIFtS benefits in significant ways from its being embedded within GeneCards. As of the introduction of GeneCards version 3 beta in May 2009, it allows advanced searches that combine gene category and data section (e.g. expression, pathways, disorders) with GIFtS range. Furthermore, GIFtS enjoys the capacities of the GeneCards batch query engine, GeneALaCart, whereby the user may receive tables with numerous genes, along with their GIFtS scores and a variety of annotation items, including pubmed IDs. Such queries can be keyed by HGNC official gene symbols or by several alias types, including Entrez Gene IDs (used in GCI queries), as well as Ensembl and SwissProt IDs.

The GIFtS scoring concepts demonstrate a generic system for evaluating genomic annotation that could be applied to a variety of species, providing that their annotation data is derived from multiple sources. This becomes more important for the many species with only minimal annotation, and in the context of the current development of high throughput, extremely rapid DNA sequencing.

Currently GeneCards focuses only on human genes, but it is rich in annotation data derived from other species., In addition to an integrated ortholog section, it has significant data from certain mammals, especially mouse, where we include in the function section mouse phenotypes from Mouse Genome Informatics (MGI), based on the strong functional overlap between the species [35], as well as mouse-related reagents (such as antibodies in the proteins section). This should serve as infrastructure for the ambitious undertaking of a bona-fide extension of GeneCards to other species, allowing full-fledged application of GIFtS to such organisms. This will allow, among others, comparisons of the landscape of annotations of the human genome, having a Genome Annotation Score (GAS) of 13081 [1], to those of genomes from other species, such as *Mus musculus *(GAS = 5644), as well as less studied species such as *Bos Taurus *with GAS<4000.

Two projects currently running in our laboratory provide clear examples for the utility of GIFtS. One is GeneDecks [36,37], a relatively new member of the GeneCards suite. One of its tools, Set Distiller, aims to detect common attributes for a set of genes. The use of GIFtS enables the profiling of gene sets, and helps choose the proper GIFtS-matched control sets for validation. The second project scrutinizes protein interaction networks in the realm of synthetic lethality [38]. The GIFtS score of each gene was used to measure the confidence in the known protein interactions for that gene, inferring an enhanced accuracy of known protein interactions for genes with a higher GIFtS score. This allows one to generate a weighted protein interaction network where high-confidence interactions receive high weights.

Further, as exemplified here, we believe that understanding the GIFtS profiles for lists of genes that result from transcription profiling experiments may help experimental biologists choose or eliminate gene candidates for future research. Finally, dissections of GIFtS data could be valuable for database construction, in decisions related to the incorporation of new gene category (e.g. RNA genes) or in the selection of additional or most relevant data sources.

## Conclusion

GIFtS yields facile tools for navigating the human gene annotation landscape, which could be useful in describing trends in human genomic knowledge, performing database maintenance and refinement, and improving computational procedures for analyzing sets of genes resulting from wet-lab or computational research. In addition, GIFtS may also assist the scientific community with identification of groups of uncharacterized genes and fields of knowledge which are less studied for diverse applications, such as delineation of novel functions and charting unexplored areas of the human genome.

## Methods

The GIFtS scores presented here are based on the data in GeneCards version 2.39, comprising 52,524 gene entries, encompassing 22,690 protein coding genes, 7,776 RNA genes, and 9,300 pseudogenes. GeneCards has recently been updated, but we expect only minor changes in analyses described in this manuscript.

The 68 sources used for the generation of GIFtS are shown in Table 1S (see additional file 1). The information about the presence or absence of gene annotation in Ns = 68 sources was extracted from the GeneCards text files by GeneQArds, the GeneCards in-house quality assurance tool (available upon request) which is based on a collection of Perl programs used for data mining and statistics. We define S_ij _as a binary N_s_-dimensional matrix, whose j-th columns depicts the absence or presence of information in data source j. The GIFtS scalar value G_i _for a gene i, is defined as . Where W_j _is a source weight. For the standard GIFtS scale, all weights are set to 1; for the experimental web tool, users can change the weights for a subset of the annotations. GIFtS vectors, calculated for each of the GeneCards genes, are stored in a MySQL 5.0 database (Sun Microsystems, Santa Clara, CA) for further analyses and dissections (e.g. extraction of the data for the GIFtS distribution of each gene category). It should be noted that the maximal score depends on the maximal fraction of the total number of sources available for the highest scoring genes. When analysing source elimination for understanding the effect of overlap, we note that after eliminating 21 sources the maximal score rises from 84 to 100.

Access to modified GIFtS scores weighted by protein sub-sections, ortholog counts and publication counts is available in the GIFtS home page [4]. In order to enrich GIFtS with respect to protein data, we selected the pivotal bioinformatics source for such data, namely SwissProt [27], and dissected it into 6 sub sources: protein subunit, sub cellular location, post-translational modification, function, catalytic activity, and other. Each of these subfields received a binary score as described above, thereby increasing the GIFtS vector size by 5. To weight proteins effectively in the new vectors, the sum of the binary data was still divided by the original number of sources (with SwissProt treated as 1 source for this denominator, in spite of its sub sources contributions to the numerator). To enrich GIFtS by orthologs or publications data, we define a new differential score for each of those components, which is then added to the default GIFtS to generate an adjusted score. Specifically, the orthologs and publications differential scores for each gene are calculated as round (log_x_sum(i)), where x equals 3 for orthologs and 5 for publications, and sum(i) is the count of relevant orthologs or publications. Genes with no orthologs or publications receive a differential score of zero for the relevant component(s); differential scores rounded down to 0 (for low counts) are set to 1 to distinguish them from a state of true 0 publication or orthologs. It should be noted that in the adjusted scores, the increment due to the addition of one or more differential scores may be rather small, as the intention is to only weigh the relevant counts in, and not to make them dominate the final outcome.

Clustering of genes according to their GIFtS vector values was performed by the k-means algorithm in MATLAB 7.5 (R2008a). The statistical significance of each cluster was calculated by using the T-test function in Microsoft Excel (Microsoft, Bellevue, WA). The HGNC approval date for gene entries was downloaded from the HGNC custom download page [39]. The significance of the difference of average GIFtS between early and late approved genes was calculated by using the T-test function in Excel.

To study the degree of overlap between a pair of sources, S1 and S2, we computed the fraction of shared genes as |*G*_*s*1_∩*G*_*s*2_|/|*G*_*s*1_∪*G*_*s*2_|, where *G*_*i *_is the set of genes appearing in source *i*.

The five Microarray-based gene lists originated from the ArrayExpress database  and appearing in: [40,41].

The three keywords-based gene lists were retrieved by searching for the terms enzyme*, develop*, in the summaries section and for cancer* in the disorders section using the advanced search of GeneCards version 3 beta [28].

Sources enrichment analysis was studied on sets of genes derived from whole genome expression studies in 12 human tissues, previously performed in our lab [12]. Eight sets of genes expressed exactly in two tissues, (based on their binary expression pattern [12]) were selected for further research. The following parameters were extracted using MySQL and PHP scripts: the number of genes with data from each one of the GeneCards sources, SS (source size); the number of genes entries in GeneCards, TG (total genes); for each set, a vector consisting of the number of genes in each of the GeneCards sources was calculated, SSSS (set specific source size); the number of genes in the set, GCS (genes count in set). These parameters enabled calculating a vector of "source enrichment " for each of the eight gene sets. The "source enrichment" was calculated for each of the GeneCards sources based on the following fraction: (SSSS/SS)X(TG/GCS). The significant of each of the "source enrichments" was evaluated by Z value, based on the distance from the average value divided by the standard deviation (average and standard deviation were calculated for all eight sets).

Sources enrichment analysis was also used to compare two gene sets (with GIFtS values of 51 or greater than 74) and is available upon request.

## Abbreviations

GIFtS: GeneCards Inferred Functionality Score; SNP: Single Nucleotide Polymorphism; GAQ: GO Annotation Quality; GAS: Genome Annotation Scores; GCI: Gene Characterization Index.

## Authors' contributions

AH developed the GIFtS scores and the GIFtS web-based tool, performed most of the analyses, and wrote the bulk of the paper. AI participated in: cluster analysis, dissection of genes by statistical parameters, analyzing overlap between sources, and evaluation of the results. GS developed GeneDecks' Set Distiller, and participated in the analysis of wet-lab transcriptional profiling experiments of expression levels from different human tissues. LSA developed GeneQArds, the GeneCards in-house Quality Assurance tool used in this work. ID pivotal member in the development of the GeneCards V3 database, participated in the analysis of GCI scores and the comparison to GIFtS data. MS is the head of the GeneCards development team, contributed to the weighted scores, the evaluation of the results, and to the writing of the paper. DL is the principal investigator, helped initiate the GIFtS idea, and contributed to the evaluation of the results and to the writing of the paper. All authors read and approved the final manuscript.

## Supplementary Material

Additional file 1 Table S1- The size (number of GeneCards entries) of GeneCards sources [42] which were used for generating the GIFtS scores. Click here for file

Additional file 2 Table S2 - Pairwise analysis for the degree of overlap between all GIFtS sources. Click here for file

Additional file 3 Fig. S1 - Effect of reducing overlapping source on distribution of GIFtS Click here for file

Additional file 4 Table S3 - GIFtS sources ids. Click here for file

Additional file 5 Fig. S2 - Distribution of GIFtS for genes with specific GCI scores [43] Click here for file

Additional file 6 Fig. S3 - Distribution of GIFtS for gene sets Click here for file

Additional file 7 Table S4- Lists of genes derived from microarray experiments. Click here for file

Additional file 8 Table S5- Lists of genes derived from keywords search in GeneCards. Click here for file

Additional file 9 Table S6 - Under and over significantly-represented GIFtS sources in each of the eight gene sets (see Methods) Click here for file

Additional file 10 Table S7 - List of genes expressed in exactly two tissues. Click here for file
