# Supplementary material for: GIFtS: annotation landscape analysis with GeneCards
Source: BMC Bioinformatics. 2009 Oct 23;10:348. doi: 10.1186/1471-2105-10-348 (PMC2774327; doi:10.1186/1471-2105-10-348)
Supplement: Additional file 1 — Table S1- The size (number of GeneCards entries) of GeneCards sources [42] which were used for generating the GIFtS scores. [file 1471-2105-10-348-S1.DOC]

## Table S1 - Size of sources.

The size (number of GeneCards entries) of GeneCards sources [42] which were used for generating the GIFtS scores. The source rank is inline with the abscissa of figure 1.

| ***Source rank*** | ***Description/Acronyms*** | ***Abbreviation*** | ***Web link*** | ***Source size (genes)*** |
| --- | --- | --- | --- | --- |
| 1 | Leiden University Muscular Dystrophy pages | LEIDEN | http://www.dmd.nl/ | 19 |
| 2 | SALK Institute. Technology transfer data | SALK | [http://www.salk.edu](http://www.salk.edu/) | 19 |
| 3 | TUFTS university. Technology transfer data. | TUFTS | http://techtransfer.tufts.edu/ | 21 |
| 4 | Database of RNA. | RNADB | <http://jsm-research.imb.uq.edu.au/rnadb/default.aspx> | 48 |
| 5 | Yeda research and development. Technology transfer data Weizmann institute. | YEDA | http://www.yedarnd.com/ | 64 |
| 6 | The Human Chromosome 21 Database at the Weizmann Institute. | CROW21 | <http://bioinfo.weizmann.ac.il/crow21> | 200 |
| 7 | BAYLOR university. Integration of cancer related data. | BAYLOR | http://tyrosine.biomedcomp.com/4d.acgi | 216 |
| 8 | Computer-annotated supplement of Swiss-Prot containing EMBL and data. | TREMBL | [http://www.uniprot.org](http://www.uniprot.org/) | 456 |
| 9 | Supplier providing a family of assays for GPCRs, kinases, protein Expression and translocation. | DiscoveRx | <http://www.discoverx.com/> | 487 |
| 10 | MicroRNA DataBase. | miRBase | <http://microrna.sanger.ac.uk/sequences/index.shtml> | 506 |
| 11 | International IMmunoGeneTics Information System. | IMGT | [http://imgt.cines.fr](http://imgt.cines.fr/) | 681 |
| 12 | GeneTests, a clinical information resource relating genetic testing to the diagnosis, management, and genetic counselling of individuals and families with specific inherited disorders. | GeneTests | [http://www.genetests.org](http://www.genetests.org/) | 696 |
| 13 | A web service presenting news about biomedical research and its applications. | Doctor's guide | http://www.docguide.com/ | 737 |
| 14 | Human Olfactory Receptor Data Exploratorium | HORDE | <http://bip.weizmann.ac.il/HORDE> | 828 |
| 15 | A supplier of high performance life science reagents, peptides and antibodies. | TOCRIS | http://www.tocris.com/ | 1093 |
| 16 | Integrated resource correlating variation in human genes leads to variation in our response to drugs | PHARMGKB | [http://www.pharmgkb.org](http://www.pharmgkb.org/) | 1226 |
| 17 | Information on pseudogenes and intergenic analyses. | GPSEUDO | http://www.pseudogene.org | 1422 |
| 18 | A supplier of specialty biochemicals, with applications in signal transduction, lipid research, apoptosis, neuroscience and drug discovery. | BIOMOL | [http://www.biomol.com](http://www.biomol.com/) | 1565 |
| 19 | Human Unidentified Gene-Encoded large proteins. | HUGE | <http://zearth.kazusa.or.jp/huge> | 2127 |
| 20 | A manufacturer of biological products with two operating divisions: Biotechnology and Hematology. | R&D | http://www.rndsystems.com | 2208 |
| 21 | Human Gene Mutation Database. | HGMD | <http://www.hgmd.cf.ac.uk/ac/index.php> | 2255 |
| 22 | Genetic Association Database. | GAD | [http://geneticassociationdb.nih.gov](http://geneticassociationdb.nih.gov/) | 2268 |
| 23 | *Saccharomyces* Genome Database. | SGD | [http://www.yeastgenome.org](http://www.yeastgenome.org/) | 2564 |
| 24 | Provides life science tools, technologies and services. | Millipore | [http://www.millipore.com](http://www.millipore.com/) | 2637 |
| 25 | A supplier of biochemical and organic chemical products and kits. | SIGMA | [http://www.sigmaaldrich.com](http://www.sigmaaldrich.com/) | 3117 |
| 26 | Atlas of Genetics and Cytogenetics in Oncology and Haematology. | ATLAS | [http://atlasgeneticsoncology.org](http://atlasgeneticsoncology.org/) | 3597 |
| 27 | Human Genome Epidemiology Navigator. | HuGE Navigator | http://www.hugenavigator.net/ | 3791 |
| 28 | Genomic Information for Eukaryotic Organisms Database for more details on similar genes in other organisms. | euGenes | [http://iubio.bio.indiana.edu:8089](http://iubio.bio.indiana.edu:8089/) | 4339 |
| 29 | Kyoto Encyclopedia of Genes and Genomes. | KEGG | http://www.genome.ad.jp/kegg/ | 4632 |
| 30 | Molecular Interactions database. | MINT | <http://mint.bio.uniroma2.it/mint> | 4837 |
| 31 | Provides research tools used to the regulation of the cell cycle and DNA repair and replication. | GeneTex | http://www.genetex.com/ | 5138 |
| 32 | A catalog of genes, markers and phenotypes with many links to major data sources. | Genatlas | <http://www.dsi.univ-paris5.fr/genatlas> | 6320 |
| 33 | Cell Signalling Technology- provides discovery tools for cell signalling research, including information about pathways and phosphorylation sites. | CST | http://www.cellsignal.com/ | 7280 |
| 34 | A database of expression variation in blood leukocytes in monozygotic twins and unrelated individuals. | Expoldb | <http://expoldb.igib.res.in/expol> | 7622 |
| 35 | A meta-database providing an extensive collection of hyperlinks to human gene-specific information in diverse databases available on the Internet. | GeneLynx | [http://www.genelynx.org](http://www.genelynx.org/) | 7751 |
| 36 | A database of known and predicted protein-protein interactions. | STRING | http://string.embl.de/ | 8055 |
| 37 | Supplier of antibodies. | ABCAM | [http://www.abcam.com](http://www.abcam.com/) | 8750 |
| 38 | A project that facilitates transferring information on human genes to Wikipedia article stubs. | GeneWiki | http://en.wikipedia.org/wiki/Gene_Wiki | 9661 |
| 39 | On-line Mendelian Inheritance in Man. | OMIM | <http://www.ncbi.nlm.nih.gov/entrez/query.fcgi?db=OMIM> | 10083 |
| 40 | The Alternative Splicing Database. | ASD | <http://www.ebi.ac.uk/asd> | 10878 |
| 41 | Alma Knowledge Server. | AKS | <http://www.bioalma.com/aks2> | 11708 |
| 42 | A Database of Blocks multiply aligned ungapped segments corresponding to the most highly conserved regions of proteins. | BLOCKS | [http://blocks.fhcrc.org](http://blocks.fhcrc.org/) | 11864 |
| 43 | A supplier of unique antibodies. | ABNOVA | http://www.abnova.com/ | 12450 |
| 44 | A database of protein families, domains and functional sites in which identifiable features found in known proteins can be applied to unknown protein sequences. | Interpro | <http://www.ebi.ac.uk/interpro> | 16239 |
| 45 | Mouse Genome Informatics (former MGD). | MGI | http://www.informatics.jax.org | 16821 |
| 46 | Cancer Genome Anatomy Project. | CGAP | [http://cgap.nci.nih.gov](http://cgap.nci.nih.gov/) | 18159 |
| 47 | Global classification of the proteins into hierarchical clusters. | ProtoNet | http://www.protonet.cs.huji.ac.il | 19408 |
| 48 | GENE Norrmal Tissue Expression database. | GeneNote | http://genecards.weizmann.ac.il/genenote/ | 19524 |
| 49 | Supplier of authentic full-length CDNA clones in a standard expression vector. | OriGene | [http://www.origene.com](http://www.origene.com/) | 19848 |
| 50 | Gene Ontology. | GO | http://www.geneontology.org/ | 19949 |
| 51 | A resource of curated and calculated orthologs for genes as represented by UniGene or by annotation of genomic sequences. | HomoloGene | http://www.ncbi.nlm.nih.gov/HomoloGene/ | 20091 |
| 52 | A supplier of life science tools, including RNAi, pathways, antibodies, and recombinant proteins. | InvitroGen | http://igene.invitrogen.com/iGene | 20629 |
| 53 | Offers an integrated view of the human and nematode genes as reconstructed by mRNA and EST alignments. | ACEVIEW | http://www.ncbi.nlm.nih.gov/IEB/Research/Acembly/index.html | 20926 |
| 54 | SNP comprehensive Database. | dbSNP | http://www.ncbi.nlm.nih.gov/SNP | 20935 |
| 55 | A curated protein sequence database which strives to provide a high level of annotation. | Swiss-Prot | [http://www.uniprot.org](http://www.uniprot.org/) | 22372 |
| 56 | Database Of Transcribed Sequences | DOTS | [http://www.allgenes.org](http://www.allgenes.org/) | 22634 |
| 57 | Annotation of high-density oligunocleotide arrays at the Weizmann Institute of Science, with links to [Affymetrix](http://www.affymetrix.com/) probe-sets. | GeneAnnot | http://genecards.weizmann.ac.il/geneannot/ | 23161 |
| 58 | Stanford Online Universal Resource for Clones and ESTs. | SOURCE | http://genome-www5.stanford.edu/cgi-bin/SMD/source | 23578 |
| 59 | An experimental system for automatically partitioning GenBank sequences into a non-redundant set of gene-oriented clusters. GeneCards extracts from UniGene the cluster (Hs.) number, gene symbol or title, GenBank accession, EST's clone identifier and library ID. | UniGene | http://www.ncbi.nlm.nih.gov/entrez/query.fcgi?db=unigene | 23578 |
| 60 | EST clustering method that produces gene models based on genomic alignment of mRNA and ESTs. | ECgene | http://genome.ewha.ac.kr/ECgene | 25463 |
| 61 | Hugo Gene Name Committee. | HGNC | http://www.gene.ucl.ac.uk/nomenclature/ | 25463 |
| 62 | H-Invitational Database (H-InvDB) is an integrated database of curated annotations of human genes and transcripts. | HinvDB | http://hinvdb.ddbj.nig.ac.jp/ahg-db/index.jsp | 25463 |
| 63 | Applied Biosystems, is a supplier of life science tools world-wide, including gene expression and genotyping assays and resequencing sets. | AB | http://www.appliedbiosystems.com | 25716 |
| 64 | Is an interactive web-based SNP analysis tool. | PupaSNP | http://pupasnp.bioinfo.cipf.es | 27448 |
| 65 | GENE Terra Incognita  Discovery Endeavor, is an automated system for human transcript (mRNAs & ESTs) annotation and elucidation of de-novo genes. | GeneTide | http://genecards.weizmann.ac.il/genetide/ | 28855 |
| 66 | A software system producing and maintaining automatic annotation on eukaryotic genomes, which provides identification of known human genes in the genome sequence and prediction of additional genes with supporting evidence. | Ensembl | [http://www.ensembl.org](http://www.ensembl.org/) | 36751 |
| 67 | At National Centre for Biotechnology Information, provides a single query interface to curated sequence and descriptive information about genetic loci. | NCBI EntrezGene | http://www.ncbi.nlm.nih.gov/entrez/query.fcgi?db=gene | 39905 |
| 68 | Integrates information on the human genome, with emphasis on mapping information. Mapped DNA segments, classified by categories (such as genes, EST clusters and STSs mapped by various methods) are presented on a Megabase-scale integrated map, with further links to relevant databases. | GENELOC | http://genecards.weizmann.ac.il/geneloc/ | 40965 |

* PubMed [41], GeneBank [42], and HapMap [43] are included via other sources.
