# Supplementary material for: GIFtS: annotation landscape analysis with GeneCards
Source: BMC Bioinformatics. 2009 Oct 23;10:348. doi: 10.1186/1471-2105-10-348 (PMC2774327; doi:10.1186/1471-2105-10-348)
Supplement: Additional file 3 — Fig. S1 - Effect of reducing overlapping source on distribution of GIFtS [file 1471-2105-10-348-S3.PPT]

## Slide 1
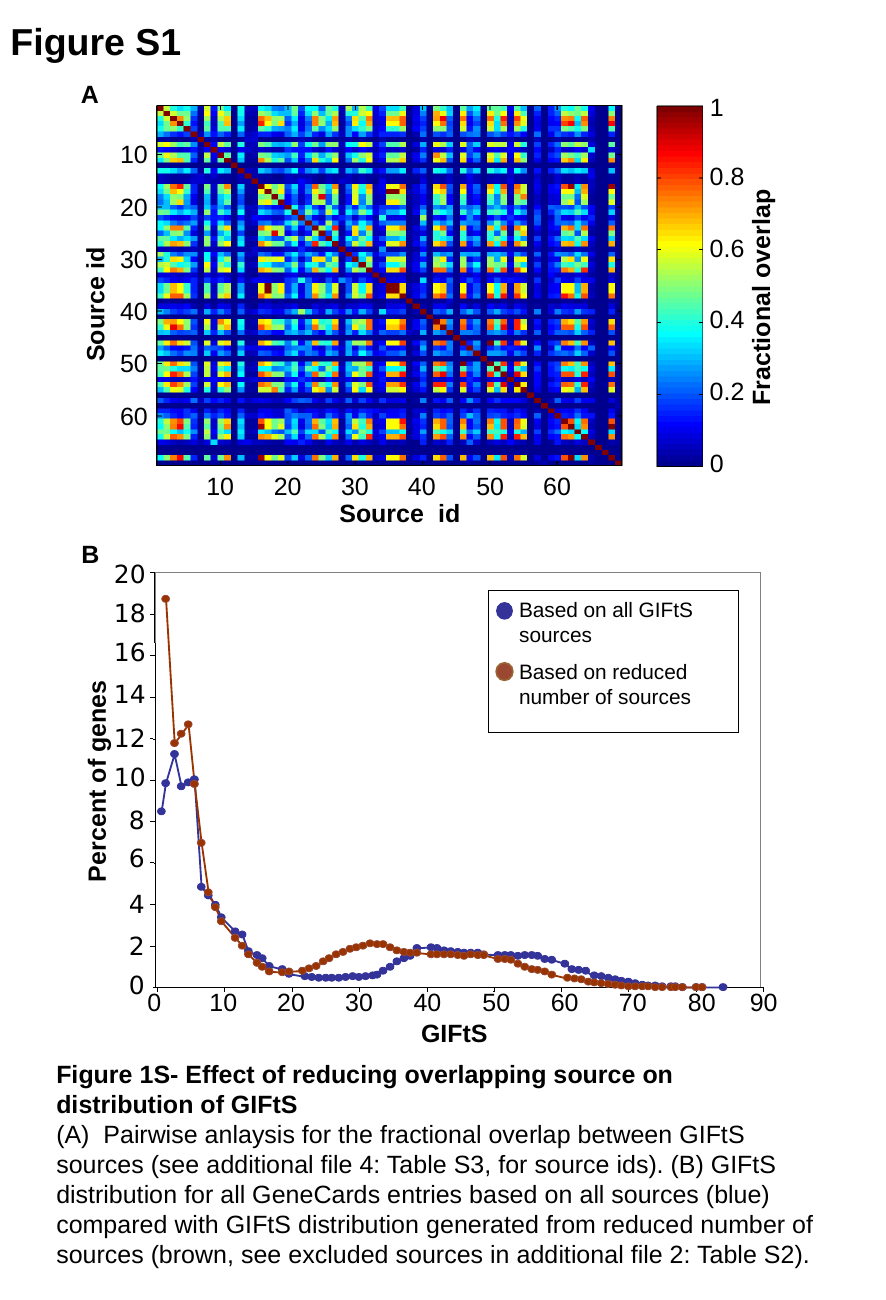

Figure S1
A
1
10
0.8
20
0.6
30
Source id
Fractional overlap
40
0.4
50
0.2
60
0
10
20
30
40
50
60
Source id
B
20
Based on all GIFtS sources
18
16
Based on reduced number of sources
14
12
Percent of genes
10
8
6
4
2
0
0
10
20
30
40
50
60
70
80
90
GIFtS
Figure 1S- Effect of reducing overlapping source on distribution of GIFtS
(A) Pairwise anlaysis for the fractional overlap between GIFtS sources (see additional file 4: Table S3, for source ids). (B) GIFtS distribution for all GeneCards entries based on all sources (blue) compared with GIFtS distribution generated from reduced number of sources (brown, see excluded sources in additional file 2: Table S2).
