# Supplementary material for: GIFtS: annotation landscape analysis with GeneCards
Source: BMC Bioinformatics. 2009 Oct 23;10:348. doi: 10.1186/1471-2105-10-348 (PMC2774327; doi:10.1186/1471-2105-10-348)
Supplement: Additional file 5 — Fig. S2 - Distribution of GIFtS for genes with specific GCI scores [43] [file 1471-2105-10-348-S5.PPT]

## Slide 1
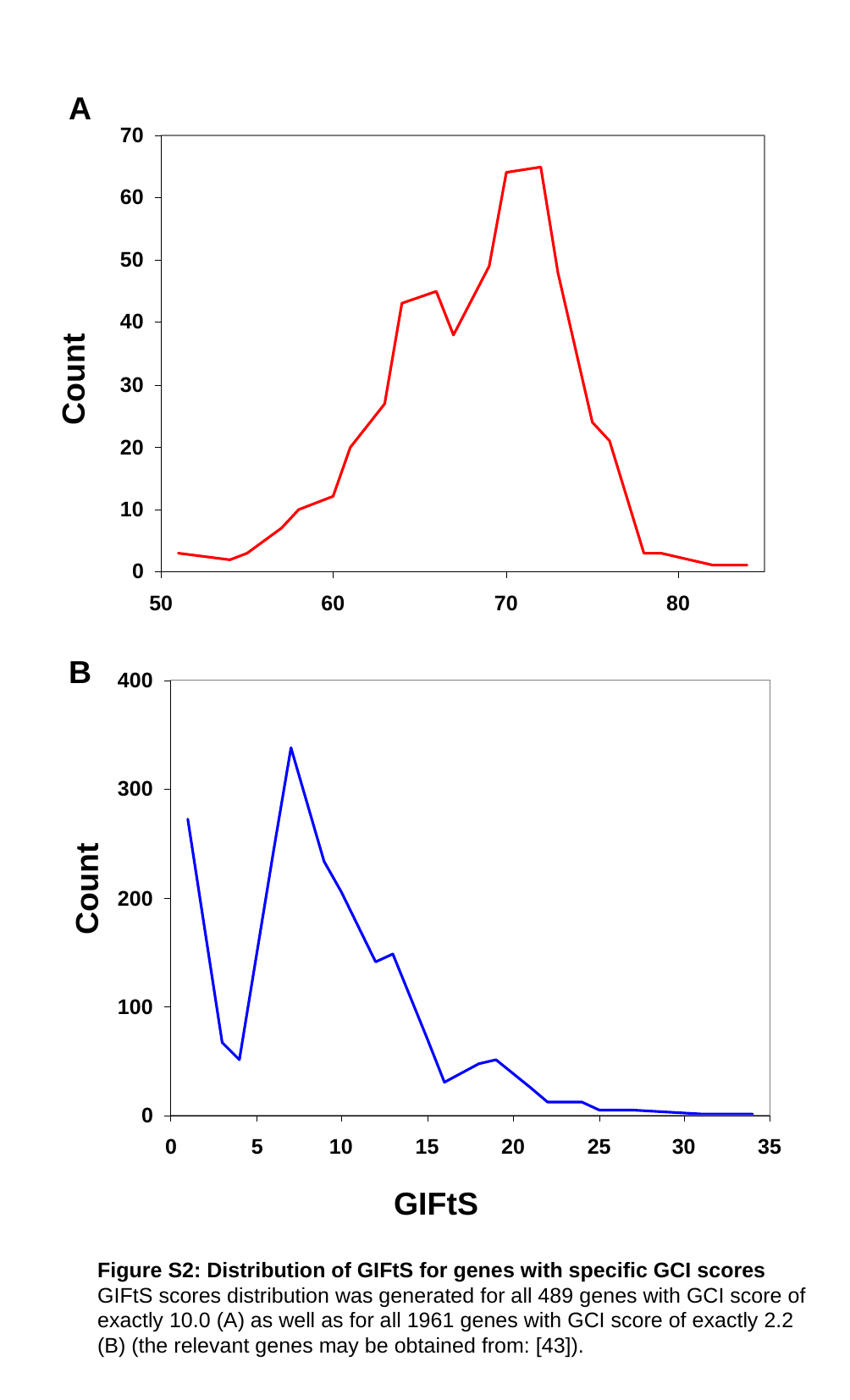

A
Count
Count
B
GIFtS
Figure S2: Distribution of GIFtS for genes with specific GCI scores
GIFtS scores distribution was generated for all 489 genes with GCI score of exactly 10.0 (A) as well as for all 1961 genes with GCI score of exactly 2.2 (B) (the relevant genes may be obtained from: [43]).
