# Supplementary material for: GIFtS: annotation landscape analysis with GeneCards
Source: BMC Bioinformatics. 2009 Oct 23;10:348. doi: 10.1186/1471-2105-10-348 (PMC2774327; doi:10.1186/1471-2105-10-348)
Supplement: Additional file 6 — Fig. S3 - Distribution of GIFtS for gene sets [file 1471-2105-10-348-S6.PPT]

## Slide 1
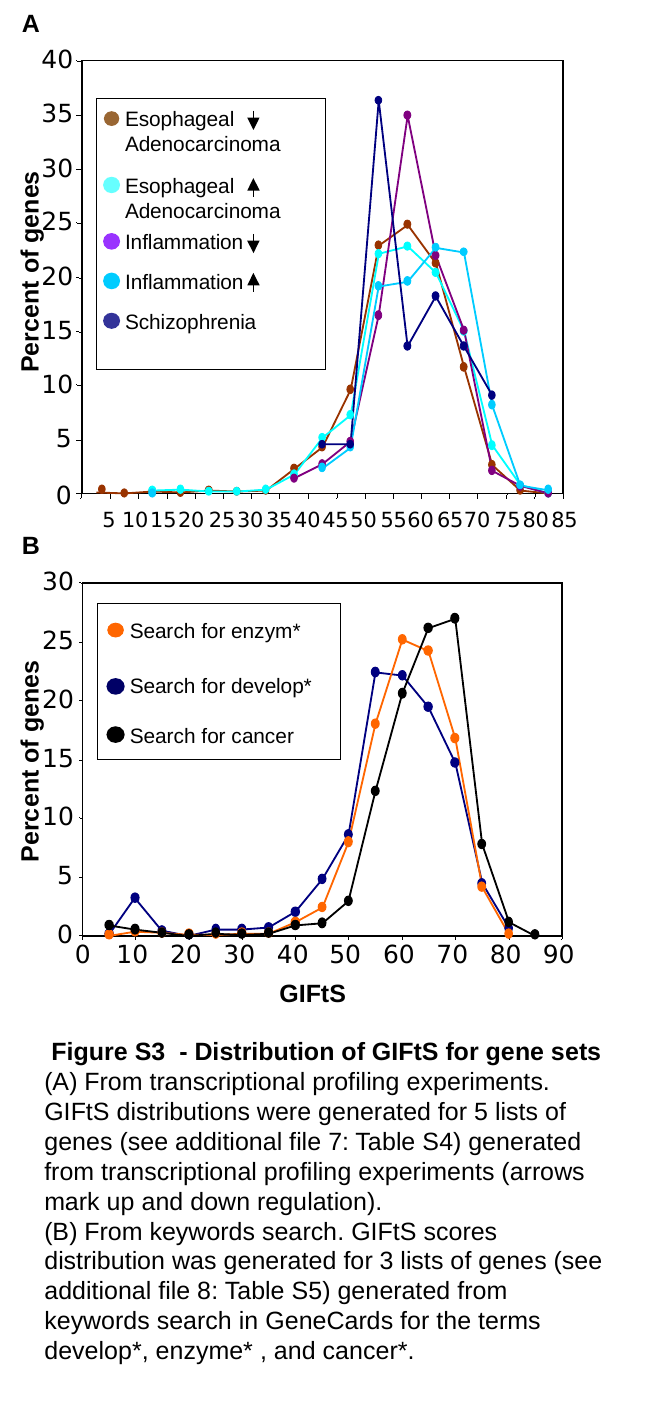

A
40
35
Esophageal
Adenocarcinoma
30
Esophageal
Adenocarcinoma
25
Percent of genes
Inflammation
20
Inflammation
Schizophrenia
15
10
5
0
5
10
15
20
25
30
35
40
45
50
55
60
65
70
75
80
85
B
30
Search for enzym*
25
Percent of genes
Search for develop*
20
Search for cancer
15
10
5
0
0
10
20
30
40
50
60
70
80
90
GIFtS
 Figure S3 - Distribution of GIFtS for gene sets (A) From transcriptional profiling experiments. GIFtS distributions were generated for 5 lists of genes (see additional file 7: Table S4) generated from transcriptional profiling experiments (arrows mark up and down regulation).
(B) From keywords search. GIFtS scores distribution was generated for 3 lists of genes (see additional file 8: Table S5) generated from keywords search in GeneCards for the terms develop*, enzyme* , and cancer*.
